# Frequencies of alleles, genotypes and haplotypes of two polymorphisms in the clusterin gene in the Russian elderly population categorized by cognitive performance

**DOI:** 10.1016/j.dib.2017.12.019

**Published:** 2017-12-16

**Authors:** Anna V. Bocharova, Kseniya V. Vagaitseva, Oksana A. Makeeva, Andrey V. Marusin, Vadim A. Stepanov

**Affiliations:** a Laboratory of Evolutionary Genetics, Institute of Medical Genetics, Tomsk National Medical Research Center, Tomsk, Russia; b Laboratory of Human Ontogenetics, Tomsk State University, Tomsk, Russia; c Nebbiolo Center for Clinical Trials, Tomsk, Russia

**Keywords:** CLU, Cognitive performance, MoCa, Russian population, Elderly, Alzheimer's disease

## Abstract

This article contains data on the frequencies of alleles, genotypes and haplotypes of the single nucleotide polymorphisms (SNPs) rs2279590 and rs1532278 in the CLU gene in a cohort of normal elderly from the Russian population. The SNPs have been reported to be associated with Alzheimer's disease and cognitive functions in genome-wide and candidate genes association studies. Cognitive performance in sample set was estimated by the Montreal Cognitive Assessment (MoCA). The frequencies of alleles, genotypes and haplotypes of two SNPs were calculated in 3 groups: total sample set, sample set with MoCA score less than 21 (the first quartile) and group with MoCA score more than 24 (the fourth quartile).

**Specifications Table**

**Table:** 

Subject area	Human Genetics
More specific subject area	Genetics of cognitive functions
Type of data	Table and figure
How data was acquired	MALDI/TOF mass spectrometry using Sequenom MassARRAY 4.0 platform (Agena Bioscience™)
Data format	Analyzed
Experimental factors	Genomic DNA was extracted from whole blood samples using phenol–chloroform extraction.
Experimental features	Genotyping of two SNPs was carried out using Sequenom iPLEX Assay following the recommended protocol by the manufacturer (Agena Bioscience™).
Data source location	Tomsk, Russian Federation
Data accessibility	The data is available within this article

**Value of the data**

• The variation in CLU gene may play a role in genetics of cognition and normal ageing.
• The data on the allele, genotype and haplotype frequencies are an important resource for understanding genetic structure of different populations.
• The frequencies of alleles, genotypes and haplotypes for rs2279590 and rs1532278 in the CLU gene in the Russian population were not previously known.
• The data can be used for comparative genetic studies of neurodegenerative diseases such as Alzheimer's disease, as well as cognitive performance in various populations.

## 1 Data

**Fig. 1:**
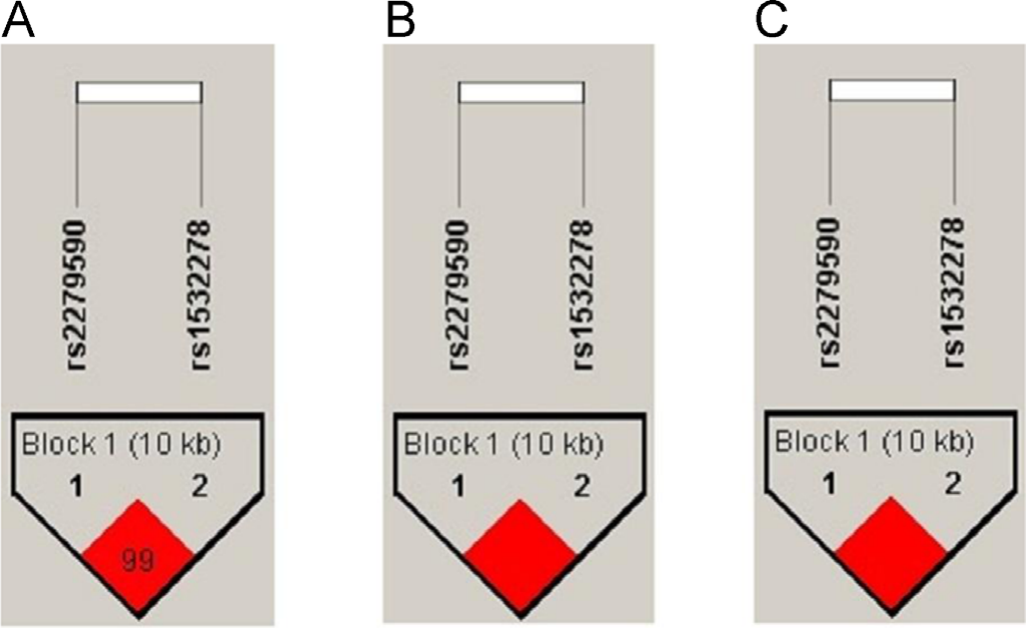
The structure of linkage disequilibrium of rs2279590 and rs1532278 in clusterin gene (CLU). Linkage disequilibrium was measured by Lewontin's coefficient *D*′. The dark red (*D*′=1) indicates that there exists strong pairwise LD between SNPs. A) All sample set (*r*^2^=0.932). B) The first quartile (total MoCA ≤ 20) (*r*^2^=0.968). C) The fourth quartile (total MoCa ≥ 25) (*r*^2^=0.916).

The data represent the frequencies of alleles, genotypes and haplotypes for single nucleotide polymorphisms (SNPs) rs2279590 and rs1532278 in human clusterin gene (CLU) associated to Alzheimer's diseases in previously published genome-wide and candidate genes association studies [1], [2], [3], [4], [5]. Russian sample set was classified into three groups according to their MoCA scores: all samples, the first quartile (total MoCA ≤ 20), the fourth quartile (total MoCa ≥ 25). The frequencies of alleles and genotypes are presented in Table 1. The description of haplotype and its frequencies are listed in Table 2.The structure of linkage disequilibrium of rs2279590 and rs1532278 in clusterin gene (CLU) is demonstrated in Fig. 1.

**Table 1:** The frequencies of alleles and genotypes for single nucleotide polymorphisms (SNPs) rs2279590 and rs1532278 in clusterin gene (CLU) in Russian elderly population.

SNP Genotype or allele	All (*n*=700)		The first quartile (total MoCA ≤ 20) (*n*=195)		The fourth quartile (total MoCa ≥ 25) (*n*=195)	
	*n*	GF or AF	*n*	GF or AF	*n*	GF or AF
rs2279590						
TT	98	0.1400	28	0.1436	24	0.1231
CT	353	0.5043	101	0.5179	103	0.5282
CC	249	0.3557	66	0.3385	68	0.3484
T	549	0.3921	157	0.4026	151	0.3872
C	851	0.6079	233	0.5974	239	0.6128

rs1532278						
TT	91	0.1300	26	0.1333	22	0.1128
CT	350	0.5000	102	0.5231	99	0.5077
CC	259	0.3700	67	0.3436	74	0.3795
T	532	0.3800	154	0.3949	143	0.3667
C	868	0.6200	236	0.6051	247	0.6333

GF, Genotypic frequency; AF, Allelic frequency.

**Table 2:** The frequencies of haplotypes for single nucleotide polymorphisms (SNPs) rs2279590 and rs1532278 in clusterin gene (CLU) in Russian elderly population.

Haplotype (rs2279590, rs1532278)	All (*n*=700) frequencies	The first quartile (total MoCA ≤ 20) (*n*=195) frequencies	The fourth quartile (total MoCa ≥ 25) (*n*=195) frequencies
CC	0.606	0.597	0.613
TT	0.378	0.395	0.367
TC	0.014	0.008	0.021
CT	0.002	–	–

## 2 Experimental design, materials and methods

### 2.1 Subjects

The study protocol was approved by the Ethics Committee of the Research Institute of Medical Genetics, Tomsk, Russian Federation. Sample of 700 elderly individuals without dementia and neurological diseases (age range 59–89 years, mean age 70.8 years) of Russian descent was randomly selected from a population-based cohort study on primary prevention of Alzheimer's disease in Tomsk, Russia [6], [7]. All of the studied individuals were Caucasians from the same ethnic (Russian) and geographical origin, living in the Tomsk region of Russian Federation. Cognitive performance was assessed using the Montreal Cognitive Assessment (MoCA) [8]. MoCA scores ranged between 0–30 points, and higher scores indicate better cognitive function. The data included 3 groups: total sample set, sample set with MoCA score less than 21 (the first quartile) and group with MoCA score more than 24 (the fourth quartile).

### 2.2 DNA extraction

Genomic DNA was extracted from the peripheral venous blood using phenol–chloroform extraction.

### 2.3 Genotyping

All 700 samples were prepared for genotyping using Sequenom iPLEX Assay following the recommended protocol by the manufacturer (Agena Bioscience™), and then were genotyped by MALDI/TOF mass spectrometry using Sequenom MassARRAY 4.0 platform (Agena Bioscience™).

### 2.4 Statistical analyses

Genotype distributions for both SNPs were in Hardy-Weinberg equilibrium, estimated by chi-square test. No significant differences in allele frequencies between the first and the forth age adjusted MoCA quartiles were found for rs2279590 (*χ*^2^=0.19, *p*=0.66) and rs1532278 (*χ*^2^=0.66, *p*=0.42). The linkage disequilibrium (LD) between rs2279590 and rs1532278 was quantified using Haploview version 4.2 software. Haplotype frequencies were determined using the EM algorithm. The LD block structure was determined using the Solid Spine of the LD algorithm [9] provided by the Haploview 4.2. The degree of genetic linkage between the 2 SNPs in 3 groups was estimated as Lewontin's coefficient D’ and Pearson's correlation coefficient r^2^, where no color (*D*′=0) indicates that LD is weak or nonexistent and the dark red (*D*′=1) indicates that there exists strong pairwise LD between SNPs (Fig. 1).

## Funding sources

The work was supported by the Russian Science Foundation (Grant no. 16-14-00020).
