# Supplementary material for: Frequencies of alleles, genotypes and haplotypes of two polymorphisms in the clusterin gene in the Russian elderly population categorized by cognitive performance
Source: Data Brief. 2017 Dec 16;16:775–9. doi: 10.1016/j.dib.2017.12.019 (PMC5738205; doi:10.1016/j.dib.2017.12.019)
Supplement: Supplementary file 1 — Supplementary material [file mmc1.docx]

**CONFLICT OF INTEREST FORM**

**Title:**

Frequencies of alleles, genotypes and haplotypes of two polymorphisms in the Clusterin gene in the Russian elderly population categorized by cognitive performance.

**Authors:**

Anna V. Bocharova, Kseniya V. Vagaitseva, , Oksana A. Makeeva, Andrey V. Marusin, Vadim A. Stepanov.

On behalf of all authors, the corresponding author of this paper declare that they have no conflict of interests.

Corresponding author:

Prof. Vadim A. Stepanov

[vadim.stepanov@medgenetics.ru](mailto:vadim.stepanov@medgenetics.ru)

Head of Laboratory of evolutionary genetics,

Institute of Medical Genetics,

Tomsk National Medical Research Center,

Tomsk, Russia
